# Monitoring the prevalence of chronic conditions: which data should we use?

**DOI:** 10.1186/1472-6963-12-365

**Published:** 2012-10-22

**Authors:** Juan F Orueta, Roberto Nuño-Solinis, Maider Mateos, Itziar Vergara, Gonzalo Grandes, Santiago Esnaola

**Affiliations:** 1 Osakidetza, Basque Health Service, C/ Alava n° 45, Vitoria-Gazteiz 01006, Spain; 2 O+berri, Basque Institute for Healthcare Innovation, Plaza Asua 1, Sondika 48150, Spain; 3 Primary Care Research Unit-Gipuzkoa, Osakidetza, P. Dr Beguiristain s/n, Instituto Biodonostia, San Sebastian, 20014, Spain; 4 Red de investigación en servicios de salud en enfermedades crónicas (REDISSEC), P. Dr Beguiristain s/n, Instituto Biodonostia, San Sebastian, 20014, Spain; 5 Asociación Centro de Excelencia Internacional en Investigación sobre Cronicidad. Kronikgune, P. Dr Beguiristain s/n, Instituto Biodonostia, San Sebastian, 20014, Spain; 6 Primary Care Research Unit-Bizkaia, Osakidetza, c/Luis Power 18 - 4ª Planta, Bilbao, 48014, Spain; 7 Department of Health and Consumer Affairs of the Basque Country, C/ Donostia-San Sebastián 1, Vitoria-Gasteiz, 01010, Spain

**Keywords:** Chronic disease, Prevalence, Information systems, Computerized medical record systems, Health care surveys, Clinical coding

## Abstract

**Background:**

Chronic diseases are an increasing threat to people’s health and to the sustainability of health organisations. Despite the need for routine monitoring systems to assess the impact of chronicity in the population and its evolution over time, currently no single source of information has been identified as suitable for this purpose. Our objective was to describe the prevalence of various chronic conditions estimated using routine data recorded by health professionals: diagnoses on hospital discharge abstracts, and primary care prescriptions and diagnoses.

**Methods:**

The ICD-9-CM codes for diagnoses and Anatomical Therapeutic Chemical (ATC) codes for prescriptions were collected for all patients in the Basque Country over 14 years of age (n=1,964,337) for a 12-month period. We employed a range of different inputs: hospital diagnoses, primary care diagnoses, primary care prescriptions and combinations thereof. Data were collapsed into the morbidity groups specified by the Johns Hopkins Adjusted Clinical Groups (ACGs) Case-Mix System. We estimated the prevalence of 12 chronic conditions, comparing the results obtained using the different data sources with each other and also with those of the Basque Health Interview Survey (ESCAV). Using the different combinations of inputs, Standardized Morbidity Ratios (SMRs) for the considered diseases were calculated for the list of patients of each general practitioner. The variances of the SMRs were used as a measure of the dispersion of the data and were compared using the Brown-Forsythe test.

**Results:**

The prevalences calculated using prescription data were higher than those obtained from diagnoses and those from the ESCAV, with two exceptions: malignant neoplasm and migraine. The variances of the SMRs obtained from the combination of all the data sources (hospital diagnoses, and primary care prescriptions and diagnoses) were significantly lower than those using only diagnoses.

**Conclusions:**

The estimated prevalence of chronic diseases varies considerably depending of the source(s) of information used. Given that administrative databases compile data registered for other purposes, the estimations obtained must be considered with caution. In a context of increasingly widespread computerisation of patient medical records, the complementary use of a range of sources may be a feasible option for the routine monitoring of the prevalence of chronic diseases.

## Background

The chronification of certain diseases and growing life expectancy of populations of most countries are leading to an increase in the number of people living with one or more chronic diseases [1,2]. Chronicity, as a wide framework for understanding the phenomenon of chronic conditions in relation to patients, their families, communities and health systems, forces us to reconsider current models of service provision and the role of patients with regard to their condition [3,4]. In relation to this, a strategy for tackling the challenges of chronicity was published in 2010 in the Basque Country [5]. It proposes medium-term changes to organise the provision of health services in an innovative way and requires the use of new information systems as a management tool when planning interventions to provide healthcare for these patients.

It is widely accepted that there is no single source of information from which we can obtain all the data necessary for routine monitoring of chronic diseases [6]. For this reason, a range of different methods are employed: demographic statistics, population surveys, specific disease registers, hospital discharge data sets and other administrative databases, as well as algorithms designed to obtain information from electronic health records (EHR) and other complex systems [7-11]. Each of these approaches has its pros and cons. In the case of administrative healthcare databases, they present as a flaw that only offer information about attended morbidity; their main advantages are that, as they contain data already recorded for other purposes, they tend to be easier to handle than other systems and are able to provide cross-sectional and longitudinal data on the prevalence and incidence of diseases in the entire population [6]. Further, in organisations such as the Spanish National Health Service, in which each patient is assigned a primary care physician and most interactions with users occur at the primary care level, the information provided by primary care health professionals may be particularly useful [7], especially since the computerization of medical offices.

The present study focuses on analysing data from routine administrative healthcare databases. As in many industrialized countries, the Basque Country has several information systems that collect data related to the healthcare of chronic conditions. First, there is a hospital discharge data system (HDDS). In addition, since 2005 the Basque Health Service, Osakidetza, extracts information from the diagnoses recorded by primary care physicians on EHRs to use in population-based case-mix systems. These data are mainly used to identify trends in morbidity seen in primary care, assess differences between geographical areas and profile health professionals. As in other places, the quality of diagnostic reporting by primary care clinicians is uneven and there is a significant degree of variability between professionals [12-14]. An alternative method for monitoring health problems is the identification of conditions from associated prescriptions. The information on medications recorded in the EHRs may be more complete and several studies have used this approach to estimate the prevalence of certain chronic diseases [15-17]. Additionally, every four years a survey on the health of the population is undertaken in the Basque Country (“Encuesta de Salud de la CAPV”, ESCAV) [18]; its findings allow us to monitor self-perceived health and the prevalence of health problems in our community as reported by the population itself.

Given the variety of data sources and the intrinsic limitations of each of them, the objective of this study was to describe the prevalence of a range of chronic diseases observed in patients under the care of the Basque health system as currently recorded in various types of administrative databases and to compare these values. To provide a context for our results from the health information systems of our region, we also report the prevalence estimated by the 2007 Basque Health Interview Survey. In addition, we observed the variations among practitioners, regarding the prevalence of disease in the populations they served, according to the different sources of information.

## Methods

This was a descriptive, cross-sectional study. The study population included all patients over 14 years of age who were assigned to a physician of the Basque Health Service (Osakidetza) for at least 6 months between 1st September 2007 and 31st August 2008 (n=1,964,337), regardless of whether they visited any doctor during this period or if at the end of the study period they maintained public insurance coverage or they had been removed for any reason (death, home moving or administrative causes).

### Setting

Osakidetza is a public healthcare organisation that provides universal coverage, funded through regional general taxation. Primary care clinicians work in teams and each person is included on the list of one physician (general practitioner [GP] if older than 14 years of age or paediatrician if younger), who acts as the gatekeeper to other levels of care. In 2008, there were 1,196 GP patient lists.

### Data sources

For this study we obtained permission from the Basque Health Service, to use the database of the Basque Country population stratification program (PREST) [19]. PREST employs an opaque identifier to ensure confidentiality of patients and contains information from hospital discharge data set (HDDS) and Osakidetza primary care EHRs. The HDDS is a set of administrative and clinical data; in our system it has been used to collect information on hospitalisation episodes since 1993 and is based on the discharge abstracts of patients admitted. Diagnoses are recorded using the International Classification of Diseases, Ninth Revision, Clinical Modification (ICD-9-CM) codes, and for this study both primary and secondary diagnoses have been taken into account.

Our EHRs were designed to facilitate the provision of individualized care to patients during their visit to a clinician. The computerisation of medical offices started in 1990 and all clinicians have had a computer in their office since 2005. The health problems of patients are organised by healthcare episodes [20] and the ICD-9-CM [21] is used as the system for coding diagnoses; this task is carried out by the primary care physicians themselves when they register or modify the diagnosis of an episode. We considered as primary care diagnoses all the ICD-9-CM codes of episodes in which any notes were entered by any GP during the observation period.

The data on prescriptions were extracted from the specific forms completed in primary care EHRs and their coding, according to the Anatomical Therapeutic Chemical (ATC) Classification System [22], was performed by the Subdepartment for Healthcare of Osakidetza.

### Identification of patients with chronic diseases

A total of 12 health problems were selected and the Adjusted Clinical Groups [23] Case-Mix System was used to identify people with each of these conditions. This system presents patient morbidity data in a range of different ways and, for this study, we focused on two of them:

– Expanded Diagnosis Clusters (EDCs): 267 groupings generated by the aggregation of ICD-9-CM coded diagnoses, according to clinical criteria. They were designed in order to enable the identification of patients with specific diseases, decreasing the differences in coding behavior between practitioners.

– Rx-Morbidity Groups (Rx-MGs): 64 groupings from a classification system based on the drugs prescribed (ATC coding system). These groups were designed according to four criteria: primary anatomical-physiological system, morbidity differentiation, expected duration, and severity of the health problem being acted by the medication.

Since the objective was to compare the prevalence of various chronic diseases as estimated from these different data sources, we selected health problems for which it is easy to establish a relationship between Rx-MGs and the corresponding EDCs. Under Spanish law, the drugs used to treat these conditions always require a doctor's prescription and can not be sold directly to patients as over the counter drugs. For some conditions there is more than one classification group, so in order to simplify the analysis data were merged. Thus, for diabetes, the four corresponding EDCs were combined, as were the two Rx-MGs, in order to create a single group of patients diagnosed with diabetes and another single group of patients receiving anti-diabetic medication; similarly, the two EDCs related to the diagnosis of arterial hypertension were grouped together.

The prevalence of the diseases in the general population was obtained from the various data sets: hospital diagnoses, primary care prescriptions and diagnoses separately and, subsequently, by combining the two sources of diagnoses (primary care and HDDS) and these with the prescription data. A patient was considered to have a medical condition if any of the diagnoses given or the drugs prescribed in the previous 12 months were included in the corresponding morbidity groups (EDCs or Rx-MGs). Since we used combined data on diagnoses and prescriptions, a patient could have both an EDC and an Rx-MG that indicate the same medical condition. Obviously, in such cases the patient was counted only once in the prevalence estimate.

### Statistical analysis

For the subpopulations assigned to each GP, prevalence rates were calculated based on three combinations of data: only primary care diagnoses, all the diagnoses (primary care and hospital) and the combination of prescription data with all the diagnoses. In order to compare the values for each GP, standardized morbidity ratios (SMRs) were calculated. The SMR is the ratio of the observed number of patients with a disease to the expected number obtained by indirectly adjusting for age and sex the overall prevalence data across Osakidetza. A value above 1.00 (for example, 1.20) indicates that the number of patients diagnosed with a specific medical condition by a given doctor is higher than the average across all the GPs in the Basque Country (in this case, 20% higher); the opposite being true for values under 1.00. A detailed description of this process for EDCs can be found in the literature [24].

In order to evaluate the dispersion of the data, namely the spread of the three SMR values for each medical condition, the variances were calculated and their differences were assessed using the Brown-Forsythe test for homogeneity of variances.

## Results

Of the population analysed, 51% were women (n=1,006,793) and 22% (n=422,719) were 65 or older. Overall, there were 188,051 admissions to hospital and 10,463,390 primary care visits. A total of 70% of patients (1,381,383) visited their GP at least once during the 12-month study period and 7% (140,737) required one or more admissions. From the HDDS we retrieved 664,185 diagnoses and from primary care EHRs 18,586,949 diagnoses and 22,518,451 prescriptions.

Table 1 shows the prevalence of the medical conditions studied in the general population by data source. The estimations obtained from the prescription data were higher than those arising from the diagnoses recorded in all cases, except for malignant neoplasm. On the other hand, for migraine higher values were obtained from the survey than using the combination of prescriptions and diagnoses.

**Table 1:** Prevalence of a range of chronic diseases by data source

	**Hospital DDS (HDDS)**		**Primary health care Dx**		**Dx (combined)**		**Rx**		**Dx (combined) + Rx**		**2007 Basque Health Survey (ESCAV)**	
	**n**	**%**	**n**	**%**	**n**	**%**	**n**	**%**	**n**	**%**		**Survey description**
Hypertension	30.342	1.54%	193.449	9.85%	207.685	10.57%	321.930	16.39%	347.344	17.68%	11.60%	High blood pressure
Lipid metabolism disorder	12.757	0.65%	140.285	7.14%	149.328	7.60%	188.704	9.61%	253.458	12.90%	6.50%	High Cholesterol
Depression	3.841	0.20%	25.103	1.28%	28.080	1.43%	120.162	6.12%	127.612	6.50%	3.30%	Anxiety-Depression
Asthma	3.557	0.18%	42.953	2.19%	44.710	2.28%	118.361	6.03%	127.208	6.48%	3.80%	Asthma
Diabetes	13.452	0.68%	81.074	4.13%	84.913	4.32%	82.994	4.23%	102.355	5.21%	3.40%	Diabetes
Osteoporosis	2.134	0.11%	25.361	1.29%	26.917	1.37%	68.091	3.47%	74.371	3.79%	2.10%	Osteoporosis
Chronic heart failure	5.766	0.29%	6.785	0.35%	10.518	0.54%	62.756	3.19%	64.191	3.27%		
Hypothyroidism	2.047	0.10%	27.965	1.42%	29.147	1.48%	53.240	2.71%	63.553	3.24%	2.20%	Thyroid Problems
Malignant neoplasm	10.316	0.53%	28.820	1.47%	33.156	1.69%	19.040	0.97%	45.970	2.34%	0.8%	Cancer
Glaucoma	1.057	0.05%	14.543	0.74%	15.183	0.77%	39.047	1.99%	41.895	2.13%		
Migraine	326	0.02%	11.234	0.57%	11.463	0.58%	14.497	0.74%	20.428	1.04%	1.9%	Migraine
Parkinson´s disease	867	0.04%	3.095	0.16%	3.571	0.18%	8.214	0.42%	8.883	0.45%		

Notes:

Total study population: 1,964,337 (over 14 years of age).

Dx: Diagnoses refers to individuals who have visited their general practitioner or have been admitted to hospital and have been diagnosed at least once with one of the listed medical conditions during the 12 months of the study.

Rx: Prescriptions means that in the primary care electronic medical record there is an entry corresponding to the issuing of a prescription (during the same 12-month period) from which it is deduced that the patient has one of the listed medical conditions.

These three sources of information are not exclusive – a single patient may be detected as having one these medical conditions through hospital, primary care and prescriptions data, although only is counted once.

The health survey covered all the population, including children.

Table 2 reports the analysis of the prevalence of each of the health problems on the GP patient lists: the variances of the SMRs based only on primary care diagnoses were the highest and those based on the combination of diagnoses and prescriptions were the lowest in all cases. In five of the diseases studied, there were no statistically significant differences in variances between the two sources of diagnosis data (primary alone and the combination of primary care and hospital), though there were differences when comparing the combination of diagnoses with the combination of diagnoses and prescriptions.

**Table 2:** Variance in standardised morbidity ratios between doctor’s lists by data source

		**Mean**	**Variance**	**Interquartile range**	**Homoscedasticity**	
					**PHC Dx vs. All Dx**	**All Dx vs. Dx + Rx**
Hypertension	PHC Dx	1.02	0.15	0.46		
	All Dx	1.02	0.11	0.41	0.0009	0.0001
	Dx + Rx	1.01	0.04	0.20		
Lipid metabolism disorder	PHC Dx	1.00	0.26	0.61		
	All Dx	1.00	0.22	0.57	0.0438	0.0001
	Dx + Px	0.99	0.08	0.34		
Depression	PHC Dx	1.00	0.54	0.91		
	All Dx	1.00	0.44	0.81	0.0057	0.0001
	Dx + Rx	1.00	0.10	0.36		
Asthma	PHC Dx	1.01	0.31	0.67		
	All Dx	1.01	0.28	0.66	0.2547	0.0001
	Dx + Rx	1.01	0.12	0.42		
Diabetes	PHC Dx	1.02	0.15	0.49		
	All Dx	1.02	0.13	0.45	0.0252	0.0001
	Dx + Rx	1.01	0.07	0.34		
Osteoporosis	PHC Dx	1.00	0.38	0.74		
	All Dx	1.00	0.33	0.69	0.1063	0.0001
	Dx + Rx	0.98	0.09	0.35		
Chronic heart failure	PHC Dx	1.04	0.58	0.90		
	All Dx	1.03	0.29	0.68	0.0001	0.0001
	Dx + Rx	1.02	0.10	0.37		
Hypothyroidism	PHC Dx	0.99	0.41	0.81		
	All Dx	1.00	0.39	0.78	0.4667	0.0001
	Dx + Rx	1.01	0.17	0.51		
Malignant neoplasm	PHC Dx	1.00	0.19	0.57		
	All Dx	1.00	0.12	0.46	0.0001	0.0001
	Dx + Rx	1.00	0.07	0.33		
Glaucoma	PHC Dx	1.02	0.51	0.87		
	All Dx	1.01	0.46	0.81	0.1693	0.0001
	Dx + Rx	0.99	0.13	0.37		
Migraine	PHC Dx	1.00	0.40	0.74		
	All Dx	1.00	0.39	0.74	0.5502	0.0001
	Dx + Rx	1.00	0.20	0.52		
Parkinson´s disease	PHC Dx	1.02	0.68	1.03		
	All Dx	1.02	0.57	0.93	0.0137	0.0001
	Dx + Rx	1.01	0.29	0.57		

PHC = Primary Health Care; All Dx = PHC diagnoses + Hospital DDS; Dx + Rx = PHC diagnoses + Hospital DDS + PHC prescriptions.

**Figure 1:**
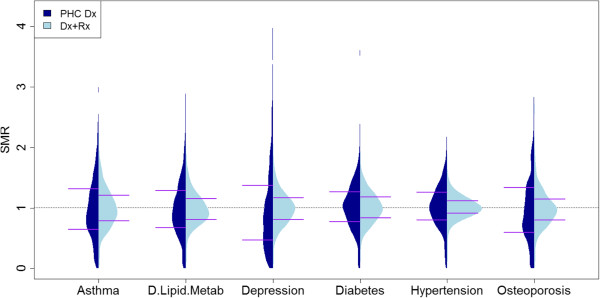
**Comparison of the distribution of the Standardized Morbidity Ratio by doctor’s lists in the medical conditions under study, by data source: primary health care diagnoses (PCH Dx) and the combination of primary care and hospital diagnoses and primary care prescriptions (Dx + Rx).** Horizontal lines correspond to values of percentiles 25 and 75.

Figure 1a and 1b show plots of the distribution of the SMRs for the GPs and illustrate the wider spread of the values obtained using the primary care diagnoses versus the combination of all of the diagnoses and prescription data. Beanplots were used rather than boxplots as, given that they show density traces of the data, they are particularly useful for comparing data coming from different groups [25].

## Discussion

Administrative databases can be a very useful tool for epidemiological surveillance: they not only enable us to estimate the incidence and prevalence of medical conditions, but also to identify cohorts of patients with a given diagnosis and follow their progression over time including the outcomes of the care they have received [26]. Nevertheless, that represents a secondary use of the data, for purposes other than that for which they were originally recorded, and the validity of the results obtained should be assessed carefully [6].

While there is no universally accepted gold standard to establish the exact prevalence of a medical condition in a given population, other studies have indicated that the detection of chronic diseases from administrative databases leads to an underestimate [6,27], especially when data used cover just one year, as in our case. The data from hospital discharge abstracts make it possible to identify chronic patients whose conditions are more severe or not under adequate control, but used alone provide limited information concerning other conditions [28] such as those considered in our study.

In our case, using different data sources we obtained inconsistent prevalence estimates. Specifically, in 11 of the 12 diseases studied prescription data indicated a larger number of chronic patients than primary care and hospital diagnoses. Further, except in the case of migraine, the combination of prescriptions and both sources of diagnoses produced higher estimates than those found in the health survey.

The differences between prevalence estimates for GP lists were smaller taking into account the combination of diagnoses and prescriptions, than using only diagnosis data. Although the distribution of medical conditions between patient populations under the care of different doctors is not random [29], the differences found in our analysis seem too large to be only attributable to disparities in the morbidity burden of the populations, especially if we consider diagnoses alone. Indeed, there are differences in the quality of the notes entered by physicians; it should be taken into account that in our health service the computerisation of patient data has been voluntary and some doctors still use paper records or prescription forms and EHRs in parallel. It cannot be proven from our study that the combination of data sources leads to a more accurate identification of patients with chronic diseases, but it seems reasonable to assume that adding pharmacy data does enable us to compensate for part of the variability attributable to the practices of doctors (in terms of entering diagnoses). We presume that flaws in physicians’ records correspond to lack of sensitivity rather than specificity and, accordingly, the simultaneous utilization of multiple data sources will notably improve sensitivity without a significant decrease of specificity.

In primary care, the completeness of data entry regarding diagnoses is influenced by a range of factors related to the organisation of the health service, the patients and the doctors themselves [17,30,31]. It should be taken into account that, in our case, the primary care data were extracted directly from the EHRs in which GPs enter notes to support the care provided to their patients; many doctors may consider it important to record the diagnosis but not necessary to enter the corresponding code. Moreover, clinicians use a wide variety of terms to describe a diagnosis, corresponding to diseases, syndromes, and symptoms, as well as complaints described by patients and other reasons for seeking medical attention [32], and often these do not conform to standard terminology. The ICD-9-MC system was originally developed for the hospital setting and its use in primary care is not straightforward. This may be particularly true in our system, since it is physicians themselves who must code diagnoses and they may consider that they are being asked to make an unnecessary effort to identify differences between terms which, in their opinion, are not clinically relevant [33]. As a result, given the lack of incentives for clinicians and the effort required, some doctors do not systematically code their diagnoses and, often (as mentioned above), leave entries concerning morbidity as free text notes only or incompletely coded [12-14]. This situation may be especially common in relation to chronic diseases such as those studied here and, indeed, it has been reported that doctors enter less accurate data on patients with multiple conditions and older patients, groups for which coding may be more challenging [17].

As we have discussed, another option for detecting health problems is prescription data [34]. Records of medication prescribed provides a list of medical conditions treated, which in some cases may be more complete than records of diagnoses; in our case, pharmacy data may be especially useful to obtain data on patients who only visit their GP for repeat prescriptions, given that their condition is stable, and do not require medical attention from their GP as their pathology is being managed through specialised outpatient services. A method based exclusively on prescriptions would not, however, be able to adequately describe the whole morbidity burden: on the one hand, there are many drugs which have numerous therapeutic uses and many medical conditions which do not have a specific pharmacological treatment and, on the other, it could lead to overestimation of prevalence among patients on the lists of doctors who tend to prescribe drugs rather than indicate other forms of treatment (e.g., nutritional advice for those with hyperlipidaemia).

The prevalence estimates we obtained using prescription data could seem too high, being bigger than those derived from survey data. In contrast, in a recent study carried out in Italy, comparing results from a range of different data sources, prevalence estimates from surveys were higher than those from pharmaceuticals prescribed for 9 out of 12 conditions studied, though the method for classifying drugs was different to the one we used [35]. In our case, the coding of the medications was carried out automatically, which minimises errors and avoids manipulation by clinicians and the classification of the drugs was performed by the robust, widely-used ACG Case-Mix System. This system recognizes that there are few drugs used to treat a single health problem; medications are assigned to categories, which can correspond to symptoms, general treatments within an organ system, and specific ones for a particular disease, trying to preserve the latter to the situations in which it has been possible to establish a 1-to-1 relation between the drug and the pathology. Further, the differences we found between prevalence estimates based on pharmacy data and on diagnoses are in line with the findings of another study carried out in a Spanish population [36]. The concordance between self-report of chronic diseases and administrative databases or registries in EHR has been studied by other authors, finding a fair-to-moderate agreement in the prevalences obtained from these sources [10,37].

Our study has certain limitations. First, although in a tax-financed insurance system that provides universal coverage, there are fewer barriers to healthcare access than in other models of care, such situation is not perfect. Thus, to the extent that some population subgroups might have a poorer access to services, their health problems would be less accurately registered. In this sense, it is not possible to rule out a certain selection bias, but this would have influenced all of data and it seems unlikely that it produced dissimilar effects on the values obtained from diagnoses, prescriptions or both sources. On the other hand, it was not possible to access the registers of Emergency Departments or specialist outpatient services; these would have provided valuable information, in particular, concerning patients who have the more severe forms of chronic conditions, or in the case of problems which are by their nature episodic, such as asthma, and others which need specialized medical care but do not require admission (for example, chemotherapy for cancer patients). In addition, we adopted relatively loose criteria, accepting that a patient presented a given condition even when there was only one recording of a diagnosis or associated prescription, so some errors due to mistakes in data entry by physicians could be overlooked. Further, the particular characteristics of our health service and the EHRs used make it difficult to extrapolate these results to other systems. Indeed, it is very difficult to compare our results with those of other studies, given substantial differences in methodology and, actually, there is not even a universally accepted definition of a chronic disease.

## Conclusions

We underline the need to be cautious when estimating the prevalence of medical conditions from data recorded for other purposes, as there may be remarkable biases in records. On the other hand, given the growing use of computers in medical offices, the complementary use of a range of data sources (in our case, diagnoses on hospital discharge abstracts, and primary care prescriptions and diagnoses) may be a feasible option to improve the accuracy of such estimations.

## Abbreviations

ACG: Johns Hopkins adjusted clinical groups case-mix system; ATC: Anatomical therapeutic chemical; EDC: Expanded diagnosis cluster; EHR: Electronic health records; ESCAV: Basque health interview survey (*Encuesta de Salud de la CAPV*); GP: General practitioner; HDDS: Hospital discarge data system; Rx-MG: Rx-morbidity groups; SMR: Standardized morbidity ratio.

## Misc

Roberto Nuño-Solinis Maider Mateos, Itziar Vergara, Gonzalo Grandes and Santiago Esnaola contributed equally to this work

## Competing interests

The authors declare that they have no competing interests.

## Authors’ contributions

All authors participated in the design of the study. JFO performed the validation of medical records. MM was responsible for statistical analyses. JFO wrote the draft of manuscript. All authors participate in interpretation of data, critically reviewed and gave final approval to the manuscript.

## Pre-publication history

The pre-publication history for this paper can be accessed here:

http://www.biomedcentral.com/1472-6963/12/365/prepub
